# Impact of Glazing, Coating, and Polishing on the Color Stability and Surface Properties of a 3D Printed Resin and Two Veneering Composite Resins

**DOI:** 10.1111/jerd.13462

**Published:** 2025-03-18

**Authors:** Marie Lask, Felicitas Mayinger, Marcel Reymus, John Meinen, Bogna Stawarczyk

**Affiliations:** ^1^ Dental Materials Unit, Department of Prosthetic Dentistry, University Hospital Ludwig Maximilian University of Munich Munich Germany; ^2^ Department of Conservative Dentistry and Periodontology University Hospital, Ludwig Maximilian University of Munich Munich Germany

**Keywords:** additive manufacturing, CIEDE2000, color, glazing, optical properties, polishing

## Abstract

**Objective:**

To analyze the impact of various surface treatments on color stability and surface properties of a 3D printed and two veneering composite resins.

**Materials and Methods:**

Specimens were manufactured from a 3D printed (VarseoSmile Crown^Plus^) or two veneering composite resins (GRADIA PLUS; VITA VM LC flow) and underwent varnishing (OPTIGLAZE; VITA AKZENT LC), coating, polishing (goat hair brush; silicone polisher) or remained untreated. For 14 days, specimens were stored in red wine, curcuma, cress, or water. Individual and, for Δ*E*_00_ > 1.8, professional prophylaxis was performed. Color (Δ*E*_00_), surface free energy (SFE), and surface roughness (SR) were measured longitudinally. Mann–Whitney *U*, Kruskal–Wallis, Friedman, and Wilcoxon tests were computed (α = 0.05).

**Results:**

For the 3D printed resin, varnishing, coating, or goat hair brushing minimized discoloration, while untreated surfaces showed the highest discoloration. Veneering composite resins benefited from goat hair brushing. Individual and professional prophylaxis improved surface properties and partially reversed discolorations. Solely goat hair brushed veneering composite resins achieved surface roughness values ≤ 0.2 μm.

**Conclusions:**

To prevent discoloration, varnishing and goat hair brushing can be recommended for all materials. Individual prophylaxis was most effective for veneering composite resin 1, whereas professional prophylaxis significantly reduced discoloration on 3D printed resin. Only veneering composite resins treated with goat hair brushing achieved surface roughness values of ≤ 0.2 μm.

**Clinical Significance:**

As 3D printed resins tend to discolor easily, it is important to understand how different surface treatments may impact their color stability. Applying treatments such as varnishing, coating, and polishing can improve the color stability and surface properties, ensuring better esthetic results over time.

## 1 Introduction

Due to recent innovations, permanent restorations can now be additively manufactured from 3D print resins and applied in a clinical setting [1, 2]. This cost‐efficient technology may represent an alternative treatment method to conventionally fabricated dental restorations, including alloy‐based and ceramic restorations. Furthermore, 3D print resins can be utilized not only for single‐tooth restorations but also as veneering materials, broadening their applications in dental prosthetics. These materials have the ability to meet the increased esthetic demands of patients who request restorations that simulate the natural tooth in color and surface texture [3]. Unfortunately, color instability and discoloration occur frequently and are reasons for replacing polymer‐based restorations such as composite resins [4, 5] and 3D printed resins [6]. Previous investigations have reported a correlation between discoloration and surface roughness: high surface roughness results in an increased surface area with different predilection sites for adhesion of color pigments [7, 8, 9]. In addition to ensuring a restoration's color stability, a low surface roughness is also needed to prevent plaque accumulation. The accumulation of plaque significantly increases the patient's risk of developing gingivitis, periodontitis, or candidiasis [10, 11]. Previous studies have shown that the color stability of a restorative material depends not only on surface roughness but also on surface free energy [9, 12]. A lower contact angle results in higher surface wettability, which in turn leads to increased adhesion of color pigments and ultimately discoloration [13]. The color stability and discoloration rate of a restoration also depend on the composition of the material, the polymerization process, and the post‐treatment of the restoration's surface [14]. Two significant factors causing discoloration can be identified: extrinsic factors from colorants of foods and drinks (e.g., red wine [9, 15], curcuma [16], or cress [17]) and intrinsic factors arising from chemical processes within the restorative material. These intrinsic factors can be examined by including control groups stored in distilled water and investigated over time [9, 18, 19]. Currently, the surface of 3D printed materials needs further improvement to meet the high standards of a homogenous, smooth, and color‐stable surface required for long‐term clinical use [20, 21]. Surface irregularities should have a smaller diameter than the average diameter of bacteria (0.2 μm), making it more difficult for them to adhere [22, 23, 24]. As of today, there are various methods to enhance the material surface after manufacturing, such as polishing, varnishing, or coating. Polymer‐based restorations can be polished using diamond finishing burs, silicone polishers, goat hair brushes, impregnated disks and cups, and different polishing pastes [10, 25, 26]. Alternatively, applying low‐viscosity glazing materials—a process termed varnishing—or unpolymerized 3D print resins—termed coating—to a restoration's surface can improve surface properties and result in a sealed, homogeneous, and smooth finish [26, 27, 28]. However, there is still a lack of investigations examining these effects for 3D printed materials [29, 30]. In clinical practice, discolorations of polymer‐based restorations can be prevented or reversed through individual or professional prophylaxis measures. Currently, only a few investigations have explored the influence of different surface treatments on the properties of 3D printed resins after storage in various media [29, 31]. To the authors' best knowledge, the potential for cleaning and reversing discolorations has also not been sufficiently studied.

Therefore, the aim of this investigation was to examine the impact of different surface treatments—namely, varnishing, coating, polishing, or no surface treatment—on the color stability, surface free energy, and surface roughness of a 3D print and two veneering composite resins after immersion in various storage media. Additionally, the investigation aimed to assess the potential of individual and professional prophylaxis measures to reverse any changes in these examined parameters.

The tested hypotheses were as follows:

1. Color deviation after 14 days would be the same for all materials regardless of surface treatment or storage medium; for all surface treatments regardless of material or storage medium; and for all storage media regardless of material or surface treatment.
2. Individual or professional prophylaxis could not improve color deviation, irrespective of the material, surface treatment, or storage medium.
3. Surface properties, namely surface free energy and surface roughness, would not be affected by different materials, surface treatments, storage media, or cleaning methods.

## 2 Material and Methods

**FIGURE 1:**
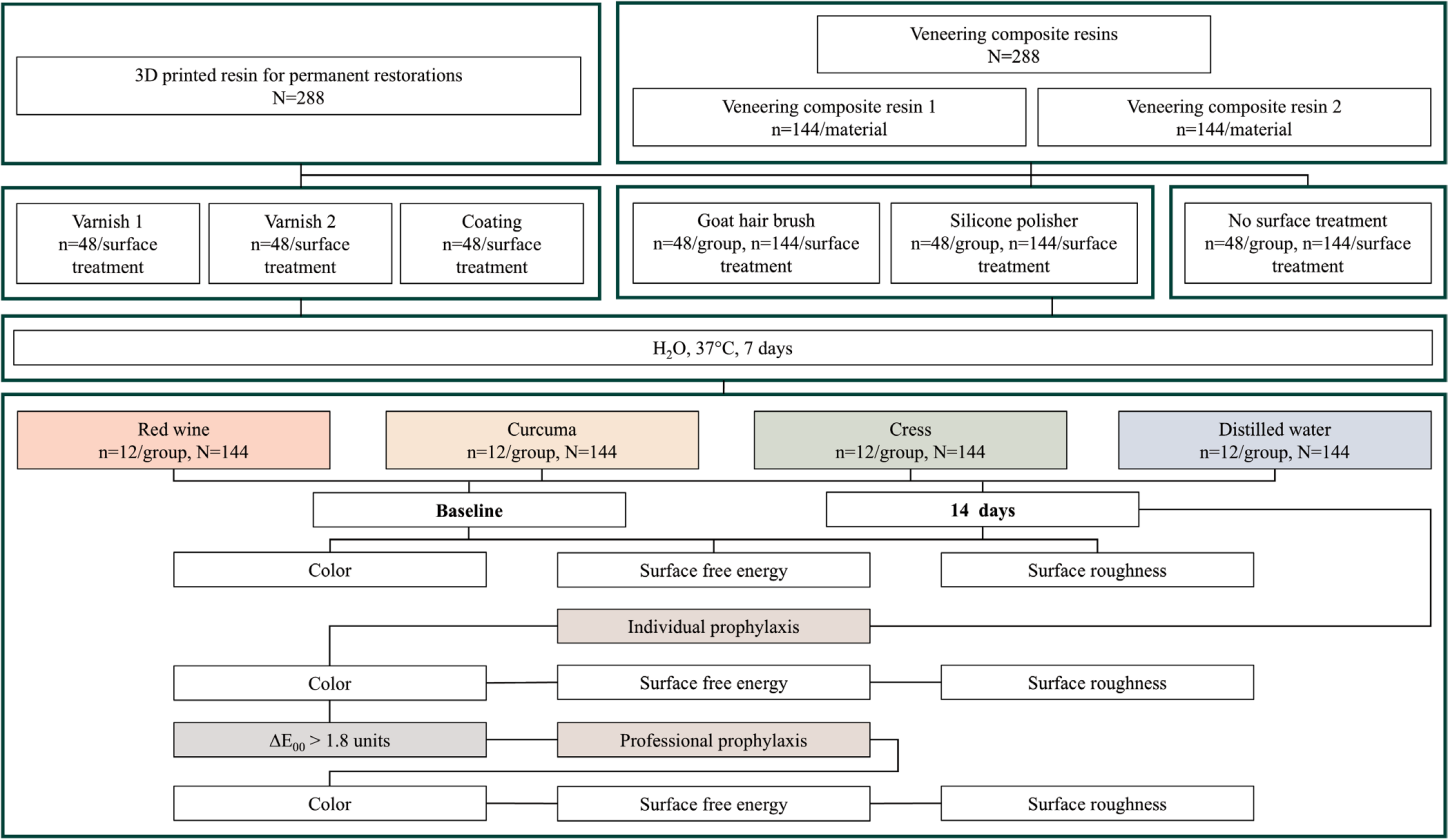
Study design.

A total of 288 specimens were additively manufactured from photopolymerizing resin (VarseoSmile Crown^Plus^, A1, BEGO, Bremen, Germany) using a 3D printer (Varseo XS, BEGO). The specimens were cleaned in an ultrasonic bath (SONOREX DIGITEC DT 31 H, Bandelin, Berlin, Germany) for 3 and 2 min with 96% ethanol (Otto Fischar, Saarbrücken, Germany) and then post‐polymerized under a nitrogen atmosphere for 2 × 1500 flashes (Otoflash G171, NK Optik, Baierbrunn, Germany) (Figure 1). Additionally, 288 specimens were made from two conventional veneering composite resins (GRADIA PLUS, GC EUROPE NV, Leuven, Belgium [veneering composite resin 1]; VITA VM LC flow, VITA Zahnfabrik, Bad Säckingen, Germany [veneering composite resin 2]). The veneering composite resins were placed in a silicone mold and light‐cured according to the manufacturers' instructions (Table 1). Specimens were then ground to achieve dimensions of a diameter of 16 mm and a thickness of 1.2 mm using 30‐μm grit silicon carbide abrasive paper (Struers Waterproof Silicon Carbide Paper FEPA P#500, Struers, Ballerup, Denmark).

**TABLE 1:** Name, technology, wavelength, polymerization time, and manufacturer of the polymerization devices used.

Name	Technology	Wavelength	Polymerization time	Manufacturer
Otoflash G171	Flashlight, nitrogen atmosphere	Spectral range 300–700 nm Peaks at 480 nm and 530 nm	2 × 1500 flashes	NK Optik
Labolight DUO	Light‐emitting diode (LED)	Spectral range 380–510 nm Peaks at 395 nm and 475 nm	Veneering composite resin 1: 2 × 3 min Varnish 1: 90 s	GC EUROPE NV
bre.Lux PowerUnit 2	Light‐emitting diode (LED)	Spectral range 370–500 nm	Veneering composite resin 2: 2 × 360 s Varnish 2: 90 s	Bredent

After manufacturing, 3D printed specimens were subjected to various surface treatments: they were either glazed with OPTIGLAZE (GC EUROPE NV [varnish 1]) or VITA AKZENT LC (VITA Zahnfabrik [varnish 2]), coated with unpolymerized resin (VarseoSmile Crown^Plus^, A1, BEGO), or polished using goat hair brushes (Rundbürste, Ziegenhaar, weiß, bredent, Senden, Germany) and polishing paste (Abraso Starglanz, bredent) or polished with silicone polishers (Sirius ceramics, Frankfurt, Germany). The varnishing and coating procedures were exclusively conducted on the 3D printed material, while polishing was applied to both the 3D printed resin and the veneering composite resins. Prior to varnishing or coating, the specimens underwent airborne particle abrasion (using 50/110 μm alumina powder, 100/150 kPa pressure, distance: 10 mm, angle: 45°, Basic quattro, Renfert, Hilzingen, Germany), followed by varnishing or coating and subsequent light polymerization (Table 1). Control groups consisted of specimens that did not receive any surface treatments. After post‐processing, specimens were stored in distilled water at 37°C for 7 days in an incubator (HeraCell 150, Heraeus, Hanau, Germany). Measurements were then performed at baseline (T0), after 14 days (T14) of media storage at 37°C in an incubator (HeraCell 150, Heraeus), and after individual and (depending on the discoloration) professional prophylaxis.

### 2.1 Media Storage

12 specimens from each group were stored in one of four different media: red wine (Cepa Lebrel 2017, Lidl, Neckarsulm, Germany), curcuma (BIO KURKUMA, EWL Naturprodukte, Ransbach‐Baumbach, Germany), cress (Bio Gartenkresse, EDEKA, Hamburg, Germany), or distilled water (Aqua Bidest, Kerndl, pH = 6.7, Vaterstetten, Germany). The storage media were replaced after 7 days. For the curcuma storage medium, 40 g of curcuma powder was mixed and dissolved in 1 L of distilled water and then boiled for 10 min. The cress medium was prepared by weighing 174 g of tamped fresh cress, which was subsequently boiled with 1 L of distilled water for 10 min and then filtered through a tea strainer to remove solid particles.

### 2.2 Individual Prophylaxis (IP)

**FIGURE 2:**
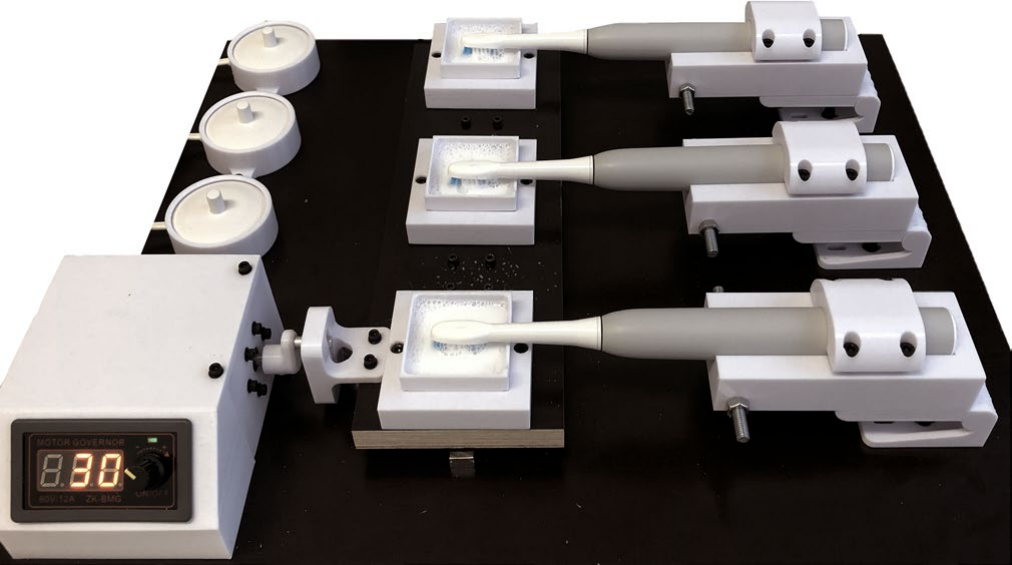
Toothbrush simulator.

After 14 days of storage in the respective media, the specimens were brushed for 4 min [9, 32] using three electronic toothbrushes (Oral‐B Pulsonic Clim Clean 2000 Gray, Procter & Gamble, Schwalbach, Germany), which were connected in series in an individually manufactured toothbrush simulator (Figure 2). To ensure that the entire surface of each specimen was adequately contacted by the bristles, the specimen holders were mounted on a movable slide rail that allowed horizontal movements at a frequency of 1 Hz. A toothpaste slurry was prepared by mixing toothpaste (Blend a Med Complete Protect Expert, Procter & Gamble) with tap water at a weight ratio of 1:2. This slurry was used during the brushing process to simulate typical oral hygiene practices.

### 2.3 Professional Prophylaxis (PP)

**FIGURE 3:**
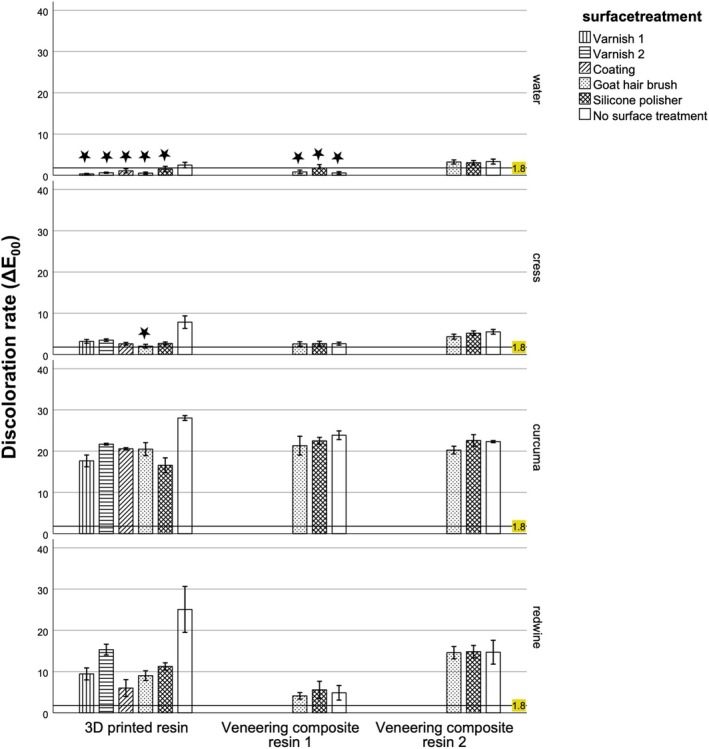
Discoloration rates after individual prophylaxis (IP); Δ*E*_00_: Baseline (T0) – IP > 1.8: Threshold for unacceptable color deviation; Δ*E*_00_ < 1.8 is highlighted (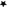).

After the individual prophylaxis, measurements were conducted to assess color, surface free energy (SFE), and surface roughness (SR). If the Δ*E*_00_ values exceeded 1.8 (Figure 3), the specimens underwent additional cleaning for 60 s using a rubber polisher and a prophylaxis paste by hand (Cleanic, KerrHawe SA, Bioggio, Switzerland) before color, SFE, and SR measurements were repeated. In color science and dental research, the acceptability threshold (AT) is set at Δ*E*_00_ = 1.8, according to ISO standards (ISO/TR 28642) [33], being particularly relevant in clinical practice [34, 35, 36].

### 2.4 Color Measurements

After media storage, specimens were cleaned in an ultrasonic bath for 3 min in distilled water. Color measurements were conducted with a spectrophotometer CM‐26dG (Konica Minolta, Tokyo, Japan). The color data software SpectraMagic NX (Konica Minolta) was used with the following parameters: standard illuminant D65, illuminating geometry d/8°, standard observer 10°, specular component included (SCI) and 100% UV. The calibration of the spectrophotometer was performed by using a white tile before every measurement cycle. The average of three measurements was recorded for each specimen in front of a white and a black background. The color parameters are listed according to the CIELAB color system (Commission Internationale de l'Eclairage, CIE), whereby L* is the lightness, a* is the green‐red, and b* is the blue‐yellow coordinate. Discolorations (Δ*E*_00_) between two different time points were calculated using the standardized formula CIEDE2000 (Δ*E*_00_).

$$\Delta E_{00}={\left[{\left(\frac{\Delta L^{\prime }}{k_{L}S_{L}}\right)}^{2}+{\left(\frac{\Delta C^{\prime }}{k_{C}S_{C}}\right)}^{2}+{\left(\frac{\Delta H^{\prime }}{k_{H}S_{H}}\right)}^{2}+R_{T}\left(\frac{\Delta C^{\prime }}{k_{C}S_{C}}\right)\left(\frac{\Delta H^{\prime }}{k_{H}S_{H}}\right)\right]}^{1/2}$$

where Δ*L*′, Δ*C*′, and Δ*H*′ represent differences in lightness, chroma, and hue; *R*_*T*_ reports on the interaction between chroma and hue differences in the blue region; *S*_*L*_, *S*_*C*_, *S*_*H*_ are weighting functions; *K*_*L*_, *K*_*C*_, *K*_*H*_ are parametric factors as correction terms for experimental conditions set to 1.

### 2.5 Surface Free Energy

**FIGURE 4:**
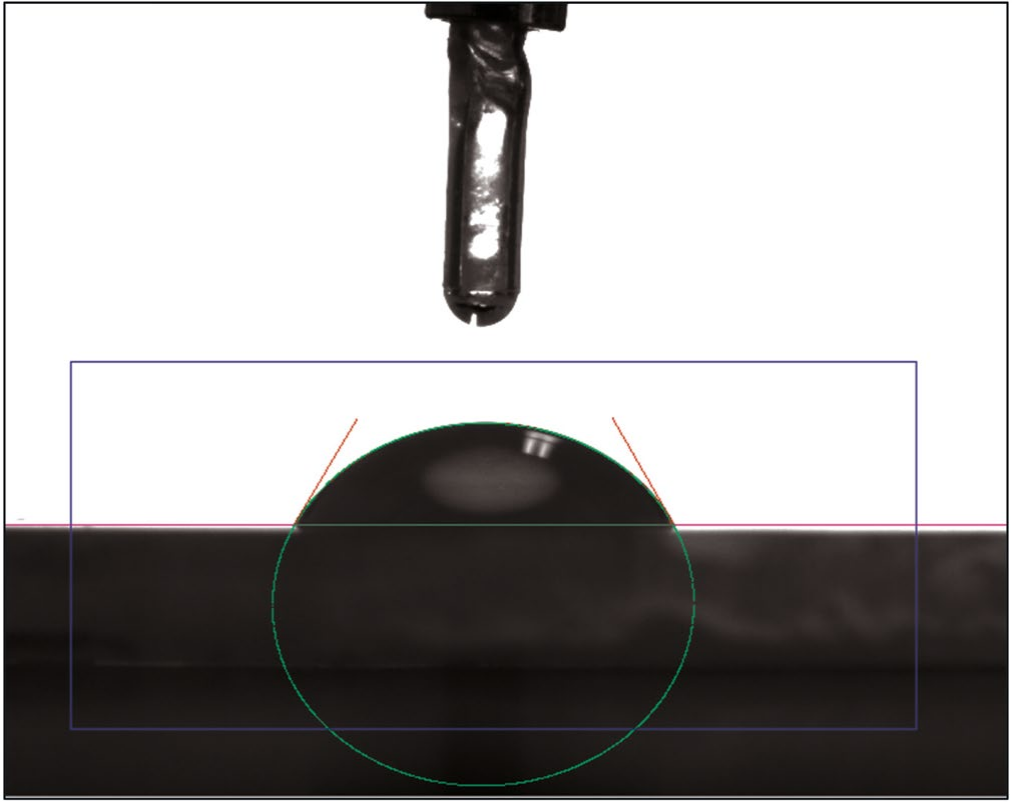
Drop shape analysis of distilled water and diiodomethane (Easy Drop, DSA4, Krüss, Hamburg, Germany).

SFE analysis was performed using the drop shape analysis system Easy Drop (DSA4, Krüss, Hamburg, Germany) through the sessile drop method. Distilled water and diiodomethane (Diiodmethane, CAS No. 75‐11‐6, Sigma–Aldrich, Bangalore, India) were applied to each specimen at room temperature. Photographs of the specimens were taken after 5 s, and the drop contour was captured using the accompanying software (Figure 4). For distilled water, the tangent‐1 method was utilized, while the circular method was employed for diiodomethane. The DSA‐4 software was used to determine surface tension by incorporating both disperse and polar fractions based on the measured contact angle and the properties of the liquid. Subsequently, SFE was calculated following the Owens‐Wendt‐Rabel‐Kaelble methodology [37].

### 2.6 Surface Roughness

SR was measured using a contact profilometer (MarSurf M400, Mahr, Göttingen, Germany) to determine the arithmetic mean roughness (Ra). For each specimen, six readings were taken—three in the horizontal and three in the vertical direction. A track length of 6 mm was used for the measurements, with 0.25 mm maintained between measurement lines.

### 2.7 Statistical Methods

Data underwent descriptive analysis, with normal distribution assessed via the Kolmogorov–Smirnov test. Non‐parametric analyses utilized the Mann–Whitney *U*, Kruskal–Wallis, Friedman, and Wilcoxon tests. Significance was inferred for *p* values < 0.05 (IBM Statistics SPSS 29.0, IBM, Armonk, USA).

## 3 Results

### 3.1 Color

As 8.3% (4/48; T0–T14), 14.6% (7/48; T14–IP) and 28.2% (11/39; IP–PP) of the Δ*E*_00_ groups deviated from the normal distribution, non‐parametric analyses were performed.

### 3.2 Δ*E*_00_ ‐1: Discoloration After 14 Days Media Storage (T0–T14)

Veneering composite resin 1 showed low Δ*E*_00_‐1 values across various treatments and storage conditions, while veneering composite resin 2 showed high values, especially after red wine, cress, or water storage (*p* < 0.001–0.018). The 3D printed resin displayed variable discoloration rates, with lower Δ*E*_00_‐1 values after specific polishing and storage combinations, such as goat hair brushing or silicone polishing with cress or water storage (*p* < 0.001–0.038) (Table 2).

**TABLE 2:** Descriptive statistics (min/med/max) for Δ*E*_00_‐1 values (T0–T14).

Material	Treatment	Red wine	Curcuma	Cress	Distilled water
3D printed resin	Varnish 1	8.59/10.5/12.3^Bx^	18.9/19.8/20.1^Aw^	2.92/3.66/4.03^By^	0.114/0.356/0.413^Az^
	Varnish 2	15.7/16.5/18.7^Dx^	20.8/21.3/22.2^Dw^	3.47/4.10/5.57^Cy^	0.393/0.506/0.600^Bz^
	Coating	4.67/6.07/9.17^Ax^	20.2/20.7/21.1^Cw^	2.33/2.59/3.04^Ay^	0.905/1.08/2.80^Dz^
	Goat hair brush	7.32/10.3/11.5^bBx^	19.7/21.0/21.8^bBCDw^	2.06/2.29/4.44*^aAy^	0.156/0.656/1.06^aCz^
	Silicone polisher	12.9/14.2/15.4^bCx^	19.7/20.5/21.6^aBw^	3.31/3.70/4.59^aBy^	0.949/1.93/2.98^aEz^
	No surface treatment	18.3/26.1/31.6^cEx^	26.1/26.9/27.3^cEx^	6.08/9.16/11.5^cDy^	0.749/1.08/2.60*^aDz^
Veneering composite resin 1	Goat hair brush	5.99/7.70/8.80^aAx^	22.0/23.0/25.0^cAw^	2.68/3.38/4.40^bAy^	1.06/1.41/2.45^bAz^
	Silicone polisher	8.15/11.9/16.3^aCx^	23.8/24.5/25.3^cBw^	3.57/4.52/5.16^bBy^	2.40/3.22/3.62^bBz^
	No surface treatment	7.27/9.03/13.0^aBx^	24.2/24.6/25.7^bBw^	3.74/4.41/5.00^aBy^	0.413/1.33/2.09^aAz^
Veneering composite resin 2	Goat hair brush	11.5/15.3/16.8^cAx^	19.0/20.2/21.5^aAw^	4.45/5.00/6.46^cAy^	2.73/3.33/4.42^cAz^
	Silicone polisher	13.8/16.9/18.4^cBx^	21.6/21.9/25.0*^bBw^	6.19/7.12/8.73^cBy^	2.75/3.82/5.38^cAz^
	No surface treatment	11.0/16.2/23.2^bABx^	21.5/22.1/22.6^aBw^	4.88/6.78/7.63*^bBy^	3.25/3.76/5.62^bAz^

*Note*: abc: differences between materials within one surface treatment and one medium storage; ABC: differences between surface treatments within one material group and one medium storage; zyxw: differences between media storages within one material group and one surface treatment.

After red wine storage, coated 3D printed resin exhibited the least discoloration, while untreated surfaces showed the most discoloration regardless of the media (*p* < 0.001). In curcuma storage, varnish 1 minimized discoloration of the 3D printed resin (*p* ≤ 0.001). The 3D printed resin showed low discoloration after cress storage with either goat hair brushing or coating (*p* < 0.001). Varnish 1 resulted in low discoloration after water storage (*p* < 0.001). For veneering composite resins 1 and 2, polishing with goat hair brushes led to low discolorations (*p* < 0.001–0.013).

For nearly all groups, water storage showed the lowest discoloration, followed by cress storage, and then by red wine storage, while the storage in curcuma resulted in the highest discoloration (*p* < 0.001).

### 3.3 Δ*E*_00_ ‐2: Reversing Discolorations After 14 Days Media Storage by Individual Prophylaxis (T14–IP)

IP achieved high stain removal for red wine, curcuma, and cress on veneering composite resin 1, with lower discoloration removal of red wine and cress on 3D printed resin and veneering composite resin 2 (*p* < 0.001–0.033) (Table 3). After cress or water storage, IP maintained strong stain removal on both veneering composites (*p* < 0.001–0.013), while 3D printed resin consistently showed low Δ*E*_00_‐2 values (*p* < 0.001–0.013).

**TABLE 3:** Descriptive statistics (min/med/max) for Δ*E*_00_‐2 values (T14–IP).

Material	Treatment	Red wine	Curcuma	Cress	Distilled water
3D printed resin	Varnish 1	0.842/1.07/1.52^Ay^	0.991/2.28/6.82^Cx^	0.789/1.01/1.21^Cy^	0.077/0.107/0.262*^Az^
	Varnish 2	1.12/1.56/3.10^BCw^	0.322/0.712/1.76^Ay^	0.749/1.10/1.89*^CDx^	0.061/0.118/0.664*^ABz^
	Coating	0.978/1.60/2.59^Bw^	0.631/1.14/1.92^Bx^	0.290/0.665/0.774*^Ay^	0.063/0.192/0.579^BCz^
	Goat hair brush	0.899/1.71/2.16^aBx^	0.623/1.15/4.78*^aBCx^	0.560/0.826/1.89*^aBy^	0.103/0.265/0.539^aCz^
	Silicone polisher	2.25/3.47/4.49^aDx^	1.30/4.76/6.73^bDw^	0.991/1.31/1.71^aDEy^	0.187/0.468/0.993^aDz^
	No surface treatment	1.28/2.17/4.56^aCy^	4.35/5.91/8.27^cEx^	1.06/1.54/2.40^bEz^	0.790/1.26/2.15^bEz^
Veneering composite resin 1	Goat hair brush	2.29/5.07/6.35^bAw^	2.22/3.92/4.40^bAx^	1.16/1.75/2.03^cAy^	0.326/0.760/1.39^bAz^
	Silicone polisher	5.38/7.29/9.32^bBw^	3.85/5.23/6.83^bBx^	2.03/2.78/3.45^bCy^	0.403/0.883/1.94^bAz^
	No surface treatment	4.02/5.81/8.58^cAw^	3.17/4.12/5.39^bAx^	1.60/1.96/2.14^cBy^	0.477/1.15/1.59^aAz^
Veneering composite resin 2	Goat hair brush	1.14/1.72/1.95^aAx^	1.00/1.78/2.39^aAx^	0.807/1.28/1.68^bAy^	0.105/0.282/0.536^aAz^
	Silicone polisher	2.30/3.46/5.57^aBx^	3.23/3.97/4.37*^aCx^	1.71/2.17/3.08^bBy^	0.419/0.843/1.40^bBz^
	No surface treatment	2.26/3.15/4.85^bBx^	1.84/2.91/3.93^aBx^	0.754/1.25/1.43^aAy^	0.275/0.753/1.58^aBz^

*Note*: abc: differences between materials within one surface treatment and one medium storage; ABC: differences between surface treatments within one material group and one medium storage; zyxw: differences between media storages within one material group and one surface treatment.

After media storage in red wine, curcuma, cress, or water, IP showed a high stain removal on the 3D‐printed resin polished with a silicone polisher or left untreated (*p* < 0.001–0.033). The application of varnish 1, varnish 2, and coating led to low stain removal (*p* < 0.001–0.043). For veneering composite resin 1 and 2 polished with a silicone polisher, the IP showed a high stain removal after red wine, curcuma, or cress storage (*p* < 0.001–0.028).

For most groups, water showed the lowest removal of discoloration, followed by cress, while red wine and curcuma showed higher values (*p* < 0.001–0.038).

### 3.4 Δ*E*_00_ ‐3: Reversing Persistent Discolorations After IP by Professional Prophylaxis (IP–PP)

For 39/48 groups (Figure 3), where Δ*E*_00_ values exceeded 1.8, indicating an unacceptable color deviation, PP was performed. For silicone polished materials, the removal of persistent red wine stains during the PP was effective on veneering composite resin 2 and the 3D printed resin (*p* < 0.001–0.011) (Table 4). For red wine, curcuma, or cress stains, the 3D printed resin and veneering composite resin 1 showed high stain removal after PP (*p* < 0.001–0.073). After water storage, the PP resulted in a high stain removal for the untreated 3D printed resin (*p* < 0.001–0.007).

**TABLE 4:** Descriptive statistics (min/med/max) for Δ*E*_00_‐3 values (IP–PP).

Material	Treatment	Red wine	Curcuma	Cress	Distilled water
3D printed resin	Varnish 1	0.665/0.899/1.30^Az^	2.56/4.33/8.05^By^	0.540/0.884/1.25^Az^	
	Varnish 2	3.55/4.84/8.89*^Cy^	6.62/8.93/13.4^Cx^	0.703/1.26/1.82^Bz^	
	Coating	0.290/0.550/1.35^Az^	3.16/9.14/17.1^Cx^	1.04/1.83/2.94^Cy^	
	Goat hair brush	0.421/0.941/1.82^aAz^	0.737/2.08/4.46^aAy^		
	Silicone polisher	1.32/1.93/2.60^bBz^	6.24/9.82/11.9^cCy^	1.11/1.92/3.47^aCDz^	
	No surface treatment	10.2/14.4/17.6^cDx^	2.23/3.69/8.30^aBy^	0.997/2.51/3.66^bDz^	2.31/4.08/5.04^by^
Veneering composite resin 1	Goat hair brush	0.430/0.834/2.55*^aAz^	2.06/4.33/5.74^bAx^	1.14/2.07/2.42^bBy^	
	Silicone polisher	0.574/1.43/2.92*^aBz^	5.04/6.57/7.81^bBy^	0.963/1.40/3.28*^aAz^	
	No surface treatment	0.899/1.30/3.38*^bBz^	3.78/8.52/9.85^bBx^	1.34/1.84/4.17^abBy^	
Veneering composite resin 2	Goat hair brush	0.228/0.604/2.63^aAy^	1.10/1.57/2.53*^aAx^	0.623/0.999/2.85*^aAy^	0.133/0.296/0.779^Az^
	Silicone polisher	1.66/3.25/4.41^cCx^	4.81/5.39/6.39^aCw^	1.30/1.57/2.89*^aBy^	0.219/0.418/1.06*^ABz^
	No surface treatment	0.674/1.10/3.72*^aBy^	3.30/3.67/4.57*^aBw^	1.22/1.72/2.24^aBx^	0.319/0.641/1.01^aBz^

*Note*: abc: differences between materials within one surface treatment and one medium storage; ABC: differences between surface treatments within one material group and one medium storage; zyxw: differences between media storages within one material group and one surface treatment.

After red wine storage and PP, the 3D printed resin with no surface treatment showed a high discoloration removal, followed by varnish 2 (*p* < 0.001). For curcuma stains, silicone polishing and coating resulted in a high discoloration removal, followed by no surface treatment and varnish 1 (*p* < 0.001–0.003). After PP, the stain removal of cress was promising on the untreated 3D printed resin, followed by the coated 3D printed resin (*p* < 0.001–0.028). The PP effectively removed red wine and curcuma stains on untreated or silicone‐polished veneering composite resin 1, and cress stains were also best removed from untreated or goat‐hair brushed surfaces (*p* < 0.001–0.021). For veneering composite resin 2, PP achieved high removal of red wine, curcuma, and cress stains, especially on untreated or silicone‐polished samples (*p* < 0.001–0.043). After water storage and PP, untreated veneering composite resin 2 showed high Δ*E*_00_‐3 values (*p* = 0.002).

For almost all tested groups, curcuma showed the highest discoloration removal during PP (*p* < 0.001–0.018).

### 3.5 SFE and SR

As 6.01% (11/183) of the SFE groups and 17.5% (32/183) of the SR groups deviated from the normal distribution, nonparametric analyses were performed.

**FIGURE 5:**
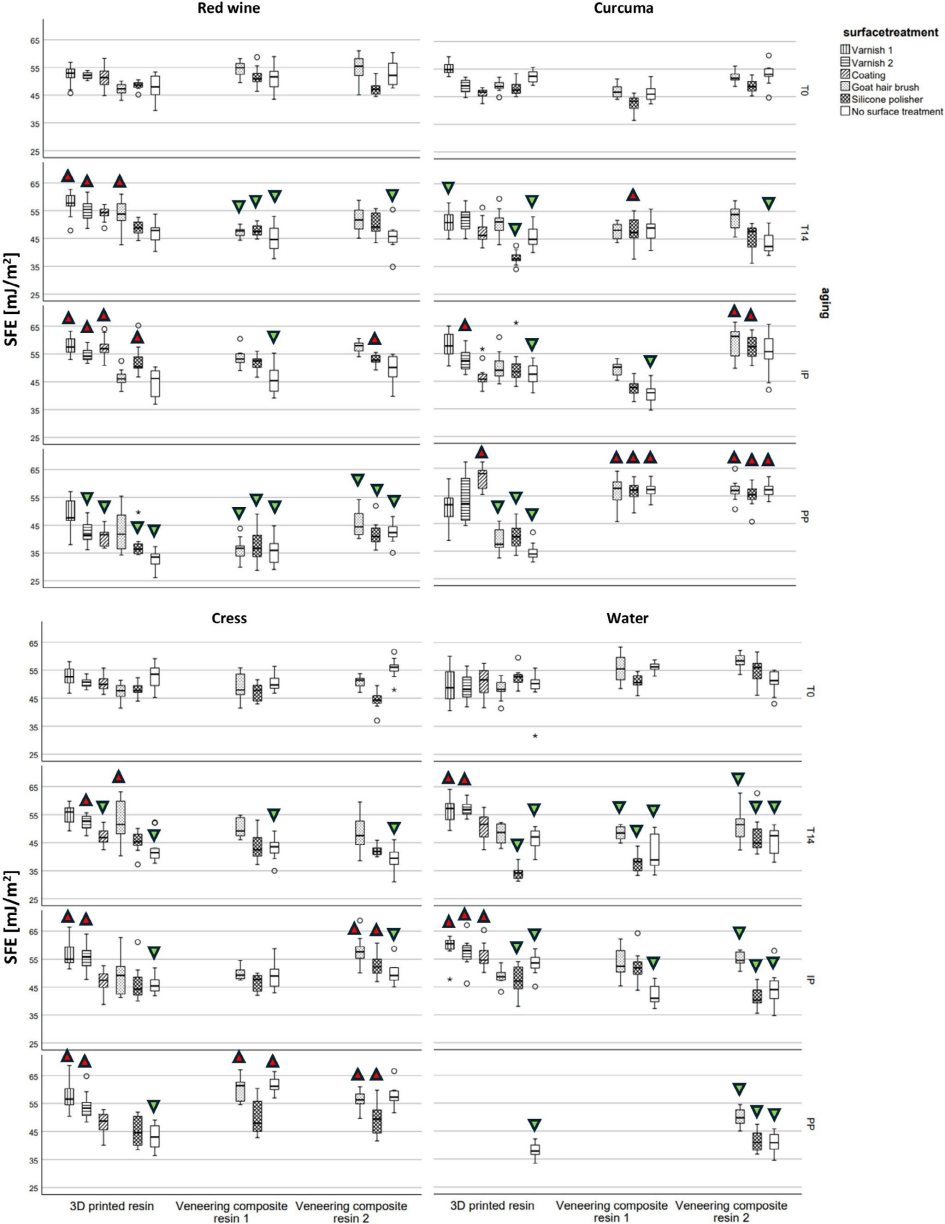
Surface free energy (SFE) in mJ/m^2^ at T0, T14, IP, and PP for the four different storage media for each group; 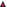 SFE increases compared to T0; 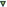 SFE decreases compared to T0.

Regarding SFE, after 14 days of storage in red wine, curcuma, cress, or water, 8/48 groups showed a significant increase in SFE (*p* = 0.002–0.023), 20/48 groups exhibited a decrease (*p* < 0.001–0.031), while all other groups (20/48) showed no significant differences (Figure 5). Examining the surface treatments for 3D printed resin, 14 days of media storage led to an increase in SFE for varnish 1, varnish 2, and goat hair brushing, while silicone polishing and no surface treatment storage resulted in a decrease (*p* < 0.001–0.031). After IP, a total of 15/48 groups experienced a significant increase in SFE (*p* < 0.001–0.024), while 11/48 groups saw a decrease (*p* < 0.001–0.042). The IP on the surfaces of varnish 1, varnish 2, and coating resulted in an increase in SFE, whereas the IP for no surface treatment led to a decrease in SFE (*p* < 0.001–0.042). After PP, a total of 13/39 groups experienced an increase in SFE (*p* < 0.001–0.031), while 18/39 groups showed a decrease (*p* < 0.001–0.007). In the cases of goat hair brushing, silicone polishing, and no surface treatment, the PP resulted in a decrease in SFE (*p* = 0.001).

**FIGURE 6:**
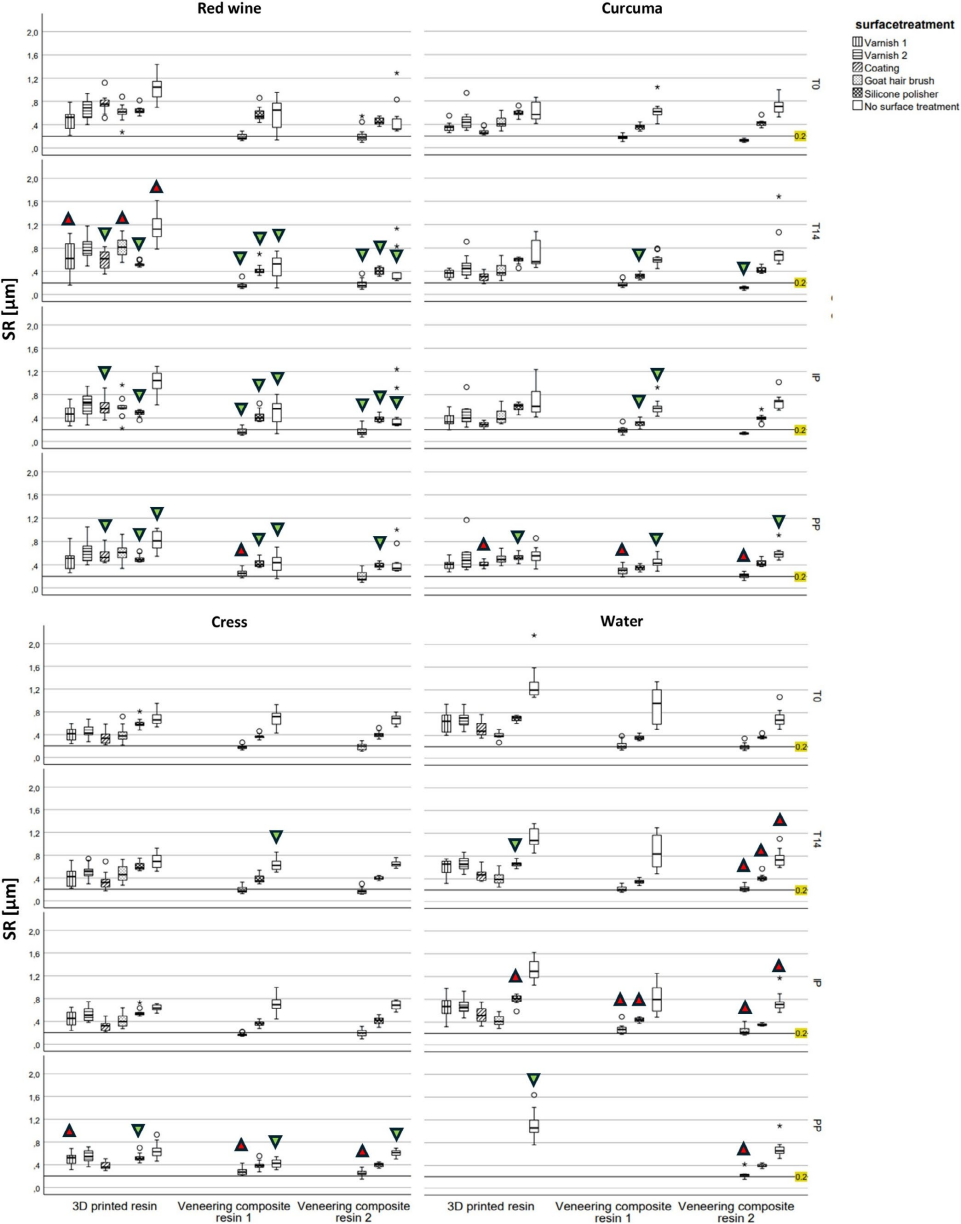
Surface roughness (SR) in μm at T0, T14, IP, and PP for the four different storage media for each group; 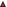 SFE increases compared to T0; 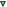 SFE decreases compared to T0.

After 14 days of storage in various media, 6/48 groups showed a meaningful increase in SR (*p* = 0.003–0.042), while 12/48 groups experienced a decrease in SR (*p* < 0.001–0.042), and 30/48 groups showed no significant differences (Figure 6). 3D printed surfaces treated with varnish 1, goat hair brush, or no surface treatment exhibited an increase in SR after media storage (*p* = 0.009–0.042). In contrast, coating and silicone polishing led to a decrease in SR (*p* < 0.001–0.023). For veneering composite resin 1, 14 days of storage resulted in a decrease in SR for surfaces that underwent goat hair brushing, silicone polishing, and no surface treatment (*p* = 0.001–0.042). Following IP, 5/48 groups exhibited an increase in SR (*p* = 0.001–0.042), and 10/48 groups showed a decrease in SR (*p* < 0.001–0.032). After IP, coated 3D printed resins showed a decrease in SR (*p* = 0.001). Veneering composite resin 1 showed a decrease in SR for surfaces that were silicone polished or received no surface treatment (*p* = 0.001–0.017). After PP, 8/39 groups had an increase in SR (*p* = 0.001–0.011), whereas 13/39 groups experienced a decrease in SR (*p* < 0.001–0.008). Varnish 1 displayed an increase in SR, while silicone polishing and no surface treatment resulted in a decrease in SR (*p* = 0.001–0.008). Veneering composite resin 2 showed an increase in SR after goat hair brushing and PP, while PP led to a decrease in silicone polished or untreated surfaces (*p* = 0.001–0.011).

## 4 Discussion

The purpose of this study was to evaluate the effects of different surface treatments (varnishing, coating, polishing, and no surface treatment) on the color stability, surface free energy, and surface roughness of a 3D printed and two veneering composite resins after immersion in various storage media. Additionally, the study aimed to determine whether individual and professional prophylaxis measures could reverse any changes observed in these parameters. As the study's findings revealed that the variables did impact color stability, surface free energy, and surface roughness in different ways depending on the conditions, the proposed hypotheses were rejected.

Although the various materials showed an impact on discoloration after 14 days of storage, the differences between the materials were not consistent. This suggests that the extent of discoloration is less influenced by the material itself and more by the type of applied surface treatment. When analyzing the effects of surface treatment, clear trends emerged: for the 3D printed resin, varnish 1, coating, or goat hair brushing resulted in the lowest overall discoloration rates. This aligns with findings from another study where a 3D printed resin glazed with GC Optiglaze and immersed in common beverages (tea, coffee, wine) demonstrated that glazing reduces surface porosity by infiltrating the material's surface and filling micropores and defects, thereby decreasing adherence of color pigments [29]. The positive outcomes associated with polishing have also been reported in previous studies investigating color stability in both 3D print resins [30] and various composite resins [7, 12]. Polishing with aluminum‐oxide or micro‐diamond polishers produced the most stain‐resistant surfaces. Coating emerged as a practical method since it requires no additional materials, making it preferable to options like varnishers. Another study indicated that coating is a more time‐ and cost‐effective alternative to conventional methods such as polishing when considering surface parameters like roughness [10]. For the 3D printed resin, it was evident that not applying any surface treatment consistently led to the highest levels of discoloration, likely due to increased surface roughness resulting from the fabrication process. This highlights the importance of applying surface treatment to 3D printed surfaces. In term of veneering composite resins, goat hair brushing resulted in the least discoloration after 14 days of storage, while silicone polishers and no treatment were less effective. The lower surface roughness achieved with goat hair brushes (≤ 0.2 μm) is likely linked to improved color stability [7, 12]. Among all tested groups, curcuma caused the most significant discoloration, followed by red wine; conversely, cress and water resulted in minimal color change. The selection of these four coloring agents was based on their established use in the literature as standard staining substances [9, 16, 17]. Foods and drinks containing pigments—such as curcuma and red wine—are known to cause pronounced staining. Curcuma's high orange pigment content leads to greater color changes compared to red wine, despite red wine's acidity and polarity enhancing surface adsorption and penetration of colorants [9, 15, 16]. Although distilled water served as a control, polymer‐based materials could still experience discoloration in water due to initiator systems like camphorquinone. The results indicated that polymer‐based materials such as 3D printed resins discolor at varying rates depending on the dietary factors. Therefore, it would be beneficial in future studies to include dietary information in patient history forms and consider this when selecting restorative materials.

It is recommended that patients adopt individual prophylactic measures, such as brushing their teeth with toothpaste, at least twice a day [32]. In this study, stains were most effectively removed from veneering composite resin 1, suggesting that this material is the easiest to clean using these prophylactic measures. Conversely, stains on the 3D printed resin and veneering composite resin 2 proved to be more resistant to individual cleaning efforts, likely due to the higher SR values of the 3D printed resin. The positive correlation between discoloration and surface roughness has been previously investigated and can be attributed to surface irregularities that create sites conducive to pigment adherence [7, 12]. The 3D printed resin without surface treatment or polished with a silicone polisher exhibited the highest stain removal rates during individual cleaning efforts. However, surfaces treated with glazing, such as varnishing or coating, showed the lowest stain removal effectiveness when subjected to IP. For both veneering composite resins, surfaces polished with a silicone polisher could be effectively cleaned using IP. These results should, however, be viewed with caution, as the surface‐treated material should not stain significantly in the first place. The cleanability of a material should thus only constitute a parameter of secondary importance. The removal of red wine and curcuma stains during the IP was more effective than the removal of cress or water stains. For water‐stored specimens, the intrinsic discoloration caused by the camphorquinone initiator system [19] could not be reversed during IP, as it cannot undo the inherent discoloration resulting from the chemical properties of the initiators within the polymers. Further studies are needed to determine why cress stains were more difficult to remove during IP than red wine or curcuma stains, even though cress groups showed overall less discoloration than red wine or curcuma.

In addition to IP measures at home, patients are advised to undergo PP measures at the dental practice at least 1 to 4 times per year [32]. PP was performed in 39 out of 48 groups (Figure 3) where Δ*E*_00_ values exceeded 1.8, indicating an unacceptable color deviation. In color assessment, the perceptibility threshold (PT) is set at Δ*E*_00_ = 0.8, marking the point at which half of the observers can detect a difference, while the acceptability threshold (AT) of 1.8 is more clinically relevant, as it defines when a color difference becomes unacceptable [35, 36]. To evaluate color changes, two common formulae are used: Δ*E*_ab_ (CIE 76) and Δ*E*_00_ (CIEDE2000), as defined by ISO/TR 28642 [33]. In this study, the CIEDE2000 formula was chosen because it more accurately represents human perception, showing a stronger correlation with visual assessments (95% for Δ*E*_00_ vs. 75% for Δ*E*_ab_) [34, 35].

The discoloration removal following PP was most effective for the 3D printed resin, followed by veneering composite resin 1. These results may be explained by the previously reported correlation between color stability, surface roughness, and surface free energy, as previous studies have shown that both surface characteristics influence the color stability of restorative materials [7, 8, 12]. In this study, the SFE and SR values for the veneering composite resins tended to increase after PP, while the values for the 3D printed resin generally decreased. Regarding the various surface treatments, untreated surfaces of the 3D printed resin were best cleaned during PP, followed by varnished (varnish 2) or coated groups. For both veneering composite resins, silicone polished or untreated surfaces were best cleaned by PP. Once again, one must consider that these surface treatments initially showed the highest discoloration rates. Through professional cleaning, stains caused by the tested beverages could, however, be partially reversed. Furthermore, studies have shown that professional cleaning can improve surface properties, such as SFE and SR, of dental materials like PEEK, PMMA, and veneering composite resins, and often help to remove discolorations [9, 32]. However, it should be noted that additional costs and time may arise for patients if they require more frequent professional cleanings that include polishing to enhance the aesthetic appearance of their prosthetic restorations.

Most of the time, the 3D printed resin showed an increase in SFE after 14 days of media storage, regardless of the surface treatment. SFE and SR are two closely correlated surface properties. As surface roughness increases, the surface area exposed to the environment also increases, which can result in a higher SFE [11]. Similarly, smoother surfaces often exhibit lower SFE. IP also led to an increase in SFE, likely due to the roughening effect caused by toothbrushes and toothpaste, which may be attributed to the abrasive nature of the toothpaste from cleaning particles. This finding aligns with results from another study, which confirmed that cleaning methods involving toothbrushes, including sonic toothbrushes, can be recommended for cleaning polyetheretherketone (PEEK) surfaces, even though they result in increased SR values compared to manual tooth brushing [32]. In contrast, PP decreased SFE and SR, likely because the use of rubber polishers and polishing paste smooths the surface, making it more homogeneous and therefore reducing SFE, as previously reported [25]. This emphasizes the inclusion of polishing all tooth surfaces in professional prophylaxis treatments to achieve surfaces that are as clean, biofilm‐free, and smooth as possible [11, 32]. Varnish 1, varnish 2, and coating resulted in higher SFE after 14 days of storage and after IP, indicating that these treatments were susceptible to surface changes during storage and the mechanical impact during IP. When comparing these results to a previous study [26], there appears to be a discrepancy in the protective effectiveness of the varnish, where varnish 2 demonstrated favorable results, with the application resulting in the lowest abrasion during three‐body‐wear. It was concluded that varnish 2 acted as an effective protective coating, limiting wear to just the varnished layer [26]. In this investigation, the same varnish could not offer the same level of protection when it came to maintaining SFE over time. However, surfaces treated with a goat hair brush, silicone polisher, or left untreated maintained stable SFE during storage and cleaning. Several studies have shown that polishing generally results in smoother and more homogeneous surfaces, improving the surface properties of materials [10, 12, 26]. This is also confirmed in the present study, as polishing the veneering composite resins, in some cases, even reduced roughness to below clinically relevant threshold values. This suggests that polishing methods, like those involving a goat hair brush or silicone polisher, create surfaces with fewer irregularities, which likely contributes to the stability of SFE. For the veneering composite resins, a significant increase (29/138 groups) or decrease (53/138 groups) in SFE and SR was observed, depending on the applied surface treatments. Interestingly, when no surface treatment was applied, PP reduced SR, which may explain the corresponding decrease in SFE. Further studies are necessary to understand why veneering composite resins exhibit different behaviors, particularly in relation to their surface properties and response to treatments.

It was noted that only veneering composite resins 1 and 2, when polished with a goat hair brush, consistently achieved the clinically relevant SR threshold of ≤ 0.2 μm. This result was typically found across all groups, regardless of media storage, storage duration, or cleaning procedures. This finding is underlined by a previous investigation showing that goat hair brushing results in the best outcomes concerning surface roughness, Martens parameters, flexural strength, and three‐body wear on veneering composite resins, indicating that this polishing system may be a better match for veneering composite resins than for 3D printed materials [26].

Previous studies have shown that the color stability of restorative materials is influenced by both surface roughness and surface free energy [7, 8, 12]. In theory, one would expect that materials with higher surface roughness and higher surface free energy would be more prone to discoloration. In this study, however, this relationship was only validated for veneering composite resins 1 and 2, which were polished with goat hair brushes. For these materials, lower surface roughness values (≤ 0.2 μm) were associated with reduced discoloration rates. Further studies are needed to investigate this finding in more detail and to determine whether it applies to other materials and polishing techniques as well.

The limitations of this investigation include the number of examined materials, surface treatments, color media, and that the aging time was set to a maximum of 14 days. As a lab‐based study, the conditions may not fully reflect clinical scenarios.

## 5 Conclusions

Within the limitations of this current study, it was concluded that:

1. For the 3D printed resin and veneering composite resins, goat hair brushing and, in the case of 3D printed resin, varnishing effectively minimized discoloration, highlighting the clinical significance of selecting appropriate surface treatments to reduce staining, especially when exposed to agents like red wine and curcuma, which caused the most staining across all materials.
2. Stains were most effectively removed from veneering composite resin 1 with individual prophylaxis, while discoloration removal after professional prophylaxis was most effective for 3D printed resin. However, material choice, surface treatment, as well as dietary preferences, is essential, as discolorations tend to persist despite undergoing prophylaxis methods.
3. With 3D printed resin, individual prophylaxis generally increased surface free energy, while professional prophylaxis decreased it. Only goat hair brushed veneering composite resins achieved surface roughness values ≤ 0.2 μm, which could be relevant for long‐term clinical outcomes, as smoother surfaces may reduce plaque accumulation and staining.

## Conflicts of Interest

The authors declare no conflicts of interest.

## Data Availability

The data that support the findings of this study are available from the corresponding author upon reasonable request.
